# Challenges in monitoring vancomycin in outpatient parenteral antimicrobial therapy: opportunities for mitigation utilizing OSHA’s framework for mitigating workplace hazards

**DOI:** 10.1017/ash.2025.10052

**Published:** 2025-06-13

**Authors:** Dominic C. Regli, Seth Warner, Ethan Wilcox, Jennifer Zimmerman, Marvin J. Bittner

**Affiliations:** 1 Creighton University School of Medicine, Omaha, NE, USA; 2 Division of Infectious Diseases, Department of Medicine, Creighton University School of Medicine, Omaha, NE, USA

## Introduction

A recent study of loss to follow-up in outpatient parenteral antimicrobial therapy (OPAT)^1^ highlighted weaknesses in transitions of care and generated a response identifying several ways to mitigate these weaknesses.^2^ These challenges have been recognized for some time, and they constitute a hazard interfering with appropriate clinical and laboratory monitoring in OPAT.^3^

We recently encountered a series of patients illustrating persistence of this hazard. We also identified several ways to mitigate this hazard. In evaluating these mitigation measures, we looked for a framework to identify the most reliable mitigation strategies. COVID-19 highlighted occupational safety concepts in infection prevention, including the concept of relative effectiveness of mitigation controls. This hierarchy of controls developed from efforts to mitigate hazards in the workplace, such as dangerous chemicals, making it appropriate for mitigating the risks of a drug with a narrow therapeutic index, such as vancomycin, in OPAT. Evaluation of various mitigation measures within the Occupational Safety and Health Administration framework may reduce the bias towards administrative controls, which are less effective and thus less appropriate for high-reliability organizations.^4^

## Report of cases

Within a six-month period, we encountered three situations where administrative problems interfered with vancomycin therapeutic drug monitoring in OPAT. In one case, an unexpectedly high vancomycin level came to the attention of an infectious diseases physician, who was unable to reach the pharmacist, home health agency, and even the patient for more than 48 hours. In a second case, an unexpectedly low vancomycin level required over 7 hours to reach the OPAT staff. In a third case, the OPAT pharmacist recognized the need for laboratory testing but could not identify the physician monitoring the patient, and the pharmacist ordered testing in the name of an uninvolved physician without telling that physician. These cases demonstrated breakdowns at multiple levels within the OPAT framework. This led to consideration of several mitigation measures.

## Hierarchy of controls

From most effective to least effective, the OSHA hierarchy of controls^4^ includes three categories relevant to reducing vancomycin hazards in OPAT: elimination, substitution, and administrative controls.

## Elimination

“Shorter is better” has become a mantra in antimicrobial stewardship.^5^ Shorter courses of vancomycin may, in some cases, be completed in the hospital. That obviates the need for vancomycin in OPAT. In other cases, shorter courses eliminate the need for therapeutic drug monitoring required.

Evidence continues to accumulate showing that shorter courses of antibiotics are adequate for types of osteomyelitis, intra-abdominal infection, and several other infections commonly managed in the OPAT setting.

Another form of elimination is replacement with well-absorbed oral drugs, requiring less extensive monitoring.^2^ A systematic review concluded that, even with risks of adverse effects, intravenous therapy could be replaced with oral therapy for “selected patients with osteomyelitis, bacteremia, and endocarditis.”^6^

## Substitution

Oritavancin and dalbavancin have spectrums of antimicrobial activity similar to vancomycin but much longer half-lives. Studies have used one dose of oritavancin or two doses of dalbavancin as an alternative to standard of care with vancomycin. These drugs don’t require the same therapeutic drug monitoring. The newer lipopeptides showed similar outcomes to standard of care and demonstrated significant cost savings across a variety of hospitalization cost levels.^7^ Daptomycin, lacking the need for routine therapeutic drug monitoring and with a better safety profile, is an additional vancomycin alternative.^3^

## Administrative controls

Despite the identification of weaknesses in transitions of care involving OPAT,^1^ expectations to mitigate this hazard are absent from the 2024 edition of the Accreditation Council for Graduate Medical Education’s guide to the Clinical Learning Environment Review, despite that publication’s specific guidance on transitions of care and teaming.^8^ One approach to mitigation of this hazard is consistent use of the I-PASS (Illness severity, Patient summary, Action list, Situation summary and contingency awareness, Synthesis by receiver) structure, which has decreased handoff errors within hospitals that implemented this framework.^9^ Using structured handoffs, such as I-PASS, for OPAT could reduce handoff errors.

Education of physicians through academic detailing and handoff training further increases reliability. These programs, through one-on-one counseling of physicians, decrease educational gaps in antibiotic usage. Academic detailing is a key part of the CDC’s Core Elements for Outpatient Antibiotic Stewardship white paper, which notes the effectiveness of academic detailing in decreasing overall antibiotic usage in other contexts.^10^

Overall, these administrative controls—while potentially effective—suffer from the same weakness attributable to administrative controls in general: dependence on consistent compliance. That is why they occupy a lower rung in the hierarchy of controls and for them to be effective, they must be paired with consistent implementation.

## Conclusions

After experiencing problems with therapeutic drug monitoring of vancomycin in OPAT, we identified several potential mitigation strategies. In identifying the most reliable strategies, we utilized OSHA’s hierarchy of controls to minimize the bias toward relatively unreliable administrative interventions. As such, we recommend consideration of interventions in this order of priority: elimination through either shorter antibiotic courses or use of oral antimicrobials, substitution of oritavancin or dalbavancin for vancomycin, and administrative controls to improve handoffs to OPAT providers and use of academic detailing to discourage inappropriate OPAT. This framework gives us a structure appropriate for high-reliability organizations to go beyond “let’s have better communications.”
